# White Matter N-Acylphosphatidylserines (NAPSs) and Myelin Dysfunction in Late-Onset Alzheimer’s Disease (LOAD): A Pilot Study

**DOI:** 10.3390/life16010022

**Published:** 2025-12-23

**Authors:** Paul L. Wood, Annika K. Lagos, Alexis R. Kastigar

**Affiliations:** Metabolomics Unit, College of Veterinary Medicine, Lincoln Memorial University, 6965 Cumberland Gap Pkwy., Harrogate, TN 37752, USA

**Keywords:** late-onset Alzheimer’s disease (LOAD), N-acylphosphatidylserines (NAPSs), N-palmitoylphosphatidylserine, N-oleoylphosphatidylserine, N-acylserines, hippocampus, periventricular white matter

## Abstract

Disruption of myelin in Alzheimer’s disease has been observed by various approaches including histology, proteomics, and white matter hyperintensities in T2 FLAIR images. Since lipids are essential myelin components, we aimed to monitor N-acylphosphatidylserines (NAPSs), unique brain lipids that are altered by neuronal stress. NAPS 52:1 (PS 36:1-N16:0) was the dominant NAPS in both gray and white matter. Relative levels of NAPS 52:1 were 2.5 times higher in the periventricular white matter (PVWM) than in the hippocampus and were reduced to approximately 50% of control in both brain regions in subjects with late-onset Alzheimer’s disease (LOAD). To monitor potential alterations in metabolic precursors of NAPS 52:1, we also measured the following: (1) phosphatidylcholine (PC) 36:1, which can undergo base exchange with N-acylserine (NASer) 16:0 to form NAPS 52:1; (2) phosphatidylserine (PS) 36:1, which can undergo N-acylation with palmitic acid (FA 16:0); and (3) diacylglycerol 36:1, which can be a precursor for both PC 36:1 and PS 36:1. These analyses found that only the relative levels of PS 36:1 were decreased and only in the PVWM. Next, we evaluated NASer 16:0, which can be released from NAPS 52:1 by phospholipase D. This is an N-acyl amino acid with neuroprotective properties. NASer 16:0 was found to be present at trace levels and could only be reliably monitored in the PVWM in which relative levels were decreased in LOAD subjects. In summary, reductions in NAPSs and NASer in the PVWM are lipid biomarkers of disruptions in myelin in LOAD. These data, in conjunction with our previous report of decrements in the levels of neocortical ether-PS in LOAD, suggest that these combined alterations in serine glycerophospholipid metabolism may contribute to neuronal dysfunction in dementia.

## 1. Introduction

Human aging is associated with a decline in cognitive function, which correlates with altered myelin function [1,2,3,4,5,6,7]. Alterations in myelin appear in imaging as white matter (WM) hyperintensities that are related to oligodendrocyte pathology involving reduced WM volume and lesions [8,9]. In LOAD, these alterations are severe and include decreased levels of myelin proteins [6,8,10] and decreased levels of myelin protein mRNAs [11]. Such findings have been further validated by single-cell transcriptomic analyses of oligodendrocytes from brains with LOAD [12]. Furthermore, studies of regional white matter hyperintensities have indicated that disruptions in the periventricular white matter (PVWM) play a key role in the manifestation of dementia in LOAD [13] and that myelin dysfunction in PVWM fiber tracts correlates with cognitive decline [1,2,4,13]. In this regard, a study of a cohort of subjects with mild cognitive impairment (MCI) found that, in individuals who progressed to AD, PVWM hyperintensities predicted this progression [14]. In addition, subcellular proteomics studies have revealed abnormal expression of an array of myelin proteins [6] including increased vesicular myelin basic protein in areas of PVWM myelin injury in subjects with LOAD [8].

While there is increasing interest in studies of myelin dysfunction in LOAD, limited lipidomics studies of white matter have been undertaken despite the fact that the lipid content of myelin is approximately 78% [15]. Studies to date of white matter from the prefrontal cortex have reported increased levels of glycerolipids and decreased levels of sulfatides [16,17] and increased levels of monogalactosyl diglycerides [16,18,19]. Recent studies of formalin-fixed brains have demonstrated that a number of lipid families can be monitored in the PVWM of fixed human brain samples [20].

With this background and data suggesting a role of endocannabinoids in neuroinflammatory mechanisms in white and gray matter [5], we aimed to perform a targeted lipidomics analysis of the PVWM and the hippocampus. The hippocampus was chosen since it is the target brain region of the septal–hippocampal cholinergic pathway [21], which is dysfunctional in LOAD [22]. In this regard, PVWM lesions result in disrupted cholinergic projections [23]. Since neuroinflammation may contribute to dysfunction in both gray and white matters [24], we undertook a study of N-acylserines, which are present in the human brain [16,25,26] and have potential neuroprotective properties [27].

In summary, neuroimaging and biochemical studies have implicated PVWM lesions as a risk for both the onset and progression of LOAD. Increasing our understanding of lipidomics alterations in the PVWM of LOAD may aid in the definition of new treatment targets for myelin maintenance/regeneration.

## 2. Materials and Methods

### 2.1. Human Brain Samples

Hippocampal and periventricular brain samples for controls and subjects with LOAD were supplied by the NIH NeuroBioBank program. The de-identified subject information is provided in Table 1. Diagnostic criteria and neuropathological assessments of the postmortem brains were conducted by clinical professionals at the academic institutions participating in the NIH NeuroBioBank program. Subjects with LOAD had disease diagnosis at greater than 70 years and demonstrated Braak stage 6 at autopsy.

### 2.2. Sample Preparation

Approximately 50 mg of brain tissue was sonicated (Thermo Fisher FB50, Waltham, MA, USA) in 1 mL of methanol and 1 mL of water containing 4 nanomoles of [^2^H_31_]phosphatiylglycerol 34:1 and 8 nanomoles of [^2^H_54_]phosphatiylcholine 28:0 (Avanti Polar Lipids, Alabaster, AL, USA). Next, 2 mL of methyl tert-butylether was added to the sonicated pellets, followed by shaking at room temperature for 30 min (Thermo Fisher Multitube Vortexer, Waltham, MA, USA) and subsequent centrifugation at 4000× *g* for 30 min at room temperature. From the upper organic layer of these centrifuged samples, 850 µL aliquots were transferred to deep-well microplates and dried via vacuum centrifugation (Eppendorf Vacfuge Plus, Thermo Fisher Scientific, Waltham, MA, USA) and stored at −20 °C. The full experimental details have already been published [16,26,28].

### 2.3. Lipidomics Analysis

To each dried sample, 200 μL of infusion solvent was added. The infusion solvent consisted of 2-propanol, methanol, and chloroform (8:4:4 ratio), containing 5 mM ammonium chloride [16,26,28]. Lipids were analyzed by flow infusion analysis (FIA) with electrospray ionization (ESI). FIA at 12 µL/min was performed utilizing high-resolution (140,000 at 200 amu) data acquisition with an orbitrap mass spectrometer (Thermo Q Exactive, Thermo Fisher Scientific, Waltham, MA, USA). The FIA 20 s scans were from 300 to 1200 amu in both positive and negative ESI modes. Between sample injections, the syringe and tubing were flushed with 1 mL of methanol followed by 1 mL of hexane, ethyl acetate, chloroform, and water (3:2:1:0.1 ratio).

### 2.4. Data Reduction

Mass spectrometric data were imported into an Excel spreadsheet containing the exact masses for monitored ions based on Table 2. The exact masses for our in-house lipid database were obtained from published lipidomics databases and peer-reviewed publications: LipidMaps [PMID 33037133], E. coli Metabolome Database (ECMDB) [PMID 26481353], Yeast Metabolome Database (YMDB) [PMID 27899612], Mycobacterium tuberculosis Database (Mtb LipidDB) [PMID 21285232], Chemical Entities of Biological Interest (ChEBI) [PMID 26467479], Human Metabolome Database (HMDB) [PMID 34986597], Seaweed Metabolite Database (SWMD) [PMID 21423723], PlanTAPDB (PMID 17337525], PubChem [PMID 33151290], and PubMed [PMID 33085945]. All our measurements are of non-oxidized lipids.

The data, which included the masses in Table 2 along with their associated peak intensities for lipids with mass errors of <2.0 ppm, were imported into a new spreadsheet. Data are presented as relative levels (Relative level = endogenous lipid peak area/peak area of internal standard normalized for protein) (Pierce BCA Protein Assay Kit; Thermo-Fisher). Individual data are presented in Supplementary Table S1.

MS^2^ validation of NAPS structures involved analysis of the product ions of precursor ions selected with a 0.4 amu window and collision energies of 25 arbitrary units. Product ions were monitored with a resolution of 140,000 (<2 ppm mass error). These included ions for the fatty acids of the phosphatidylserine (PS) and the N-acyl fatty acid, along with the phosphatidic acid of the PS [16].

Data were analyzed via the Student’s *t*-test, assuming equal variances (Excel, Microsoft, Seatle, WA, USA).

## 3. Results

### 3.1. NAPS

**Figure 1:**
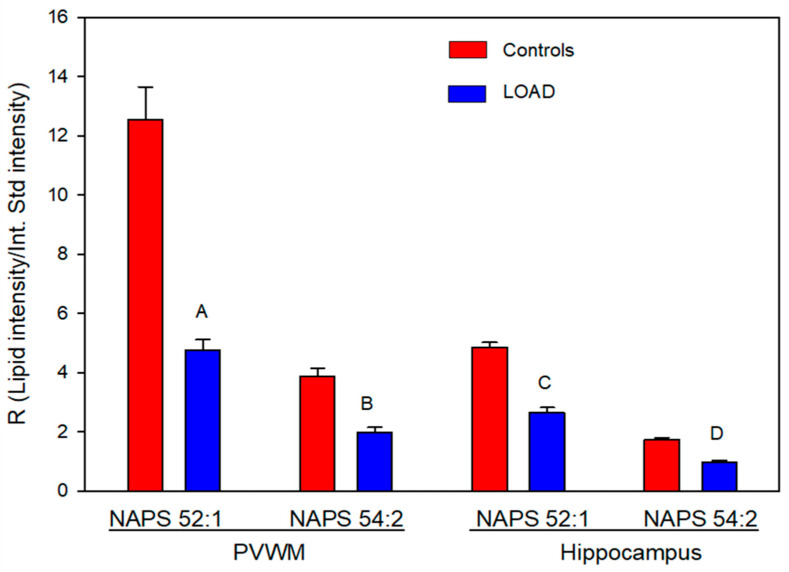
Relative levels of NAPS in the periventricular white matter (PVWM) and hippocampus of controls and subjects with LOAD. A (*p* = 2.38 × 10^−7^), B (*p* = 2.81 × 10^−7^), C (*p* = 9.06 × 10^−6^), and D (*p* = 3.56 × 10^−5^). N = 8 per group.

NAPS 52:1 was the dominant NAPS in both the hippocampus and the PVWM, as reported previously for the human frontal cortex [16,25] and the mouse brain [29]. Levels of NAPS 52:1 were approximately three times greater than those of NAPS 54:2, with the levels of both NAPSs in LOAD tissues found to be about 50% of that in controls (Figure 1). The PVWM contained the highest relative levels of NAPS 52:1. This was approximately 2.5 times the levels present in hippocampal tissue (Figure 1).

**Figure 2:**
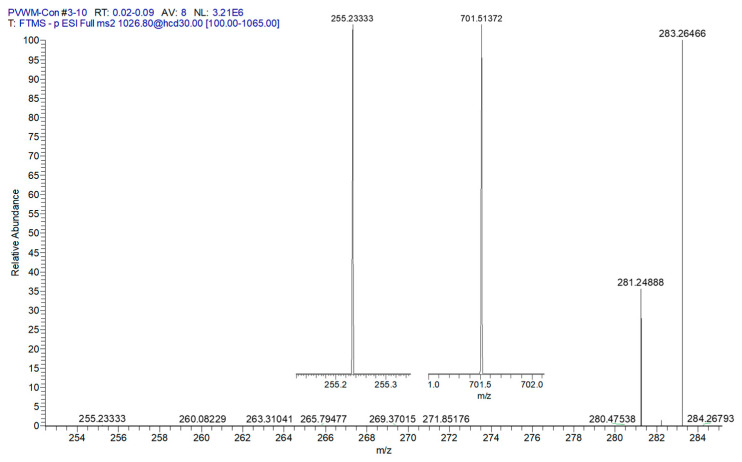
MS^2^ of PVWM NAPS 52:1 (PS 18:0/18:1;N 16:0). FA 18:0 (283.2646; 1.22 ppm), FA 18:1 (281.2488; 0.69 ppm), and inserts of FA 16:0 (255.2333; 1.37 ppm) and phosphatidic acid 36:1 (18:0/18:1 = (701.5137; 1.46 ppm). These data support the structure of NAPS 52:1 as N-16:0-PS 18:0/18:1, as previously reported for the human frontal cortex [16] and mouse brain [29].

Validation of the NAPS structures involved monitoring the three fatty acid product ions and the phosphatidic acid product ion to deduce which fatty acid was the N-acyl substitution [16]. The MS^2^ analyses (Figure 2) resulted in product ions (<2 ppm mass error) for phosphatidic acid (PA) 36:1 and fatty acids 16:0, 18:0, and 18:1. These product ions indicate that the PA is PA 18:0/18:1 and that the N-acyl substituent is FA 16:0, supporting the structure of NAPS 52:1 as N-16:0-PS 18:0/18:1, as previously reported for the human frontal cortex [16] and mouse brain [29]. NAPS 54:2 was determined to be N-18:1-PS 18:0/18:1. These data indicate that the N-acylation of PS involves short-chain fatty acids and not long-chain or polyunsaturated fatty acids (PUFAs). Since there are a number of PUFA-containing N-acylserines in the brain, these data may support direct acylation of PS by acyltransferases.

### 3.2. Phosphatidylcholine (PC) 36:1 and Phosphatidylserine (PS) 36:1

**Figure 3:**
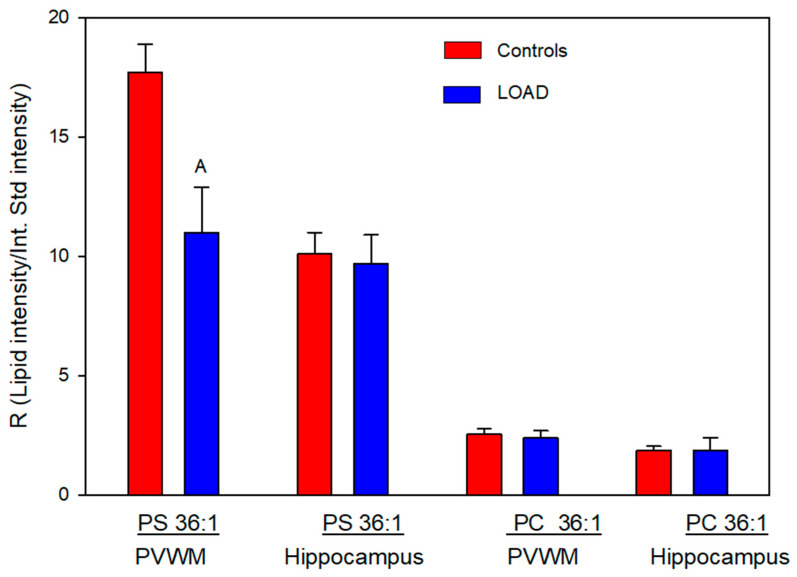
Relative levels of phosphatidylserine (PS) 36:1 and phosphatidylcholine (PC) 36:1 in the PVWM and hippocampus of controls and subjects with LOAD. A (*p* = 0.013). N = 8 per group.

**Figure 4:**
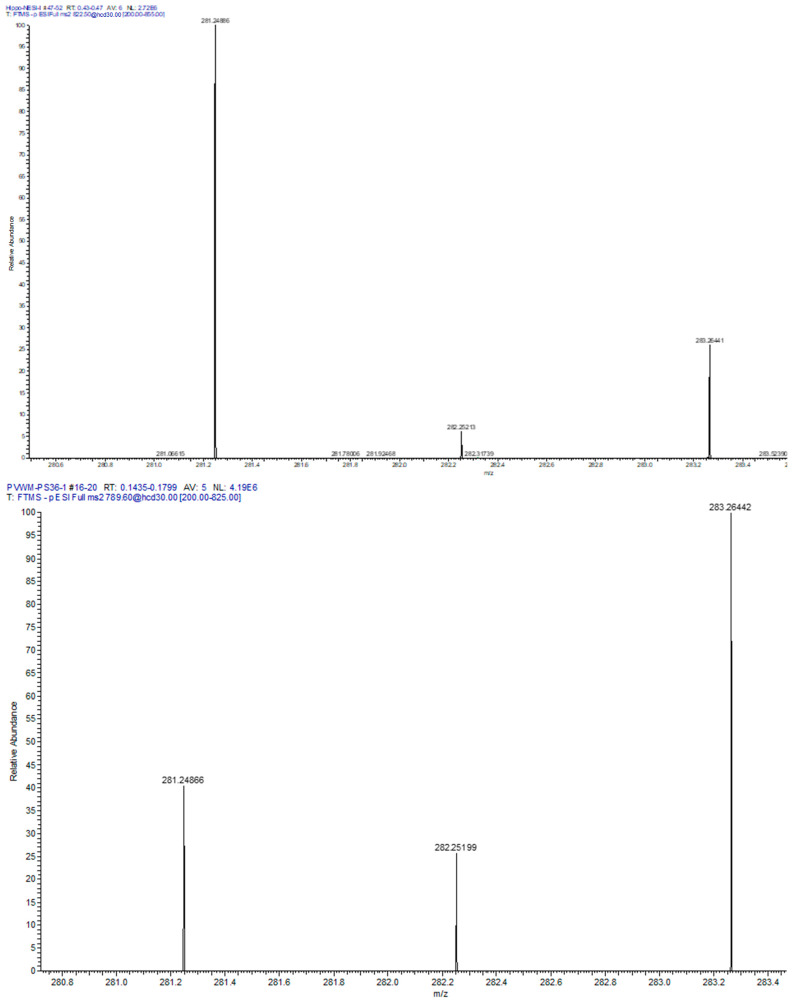
MS^2^ of PVWM PC 36:1 ([M + Cl = 822.58]^−^ (**left** spectrum) and PS 36:1 [M-H = 788.54]^−^ (**right** spectrum). In both cases the fatty acids 18:1 (281.2486, 0.69 ppm) and 18:0 (283.2642, 0.51 ppm) were monitored, supporting the structures of PC 36:1 and PS 36:1.

Next we evaluated the relative levels of glycerophospholipid putative precursors of NAPS 52:1 and 54:2. Since both NAPS 52:1 and NAPS 54:2 are N-substituted PS 36:1, this simplified our analysis to the evaluation of PC 36:1, which would involve base exchange of PC 36:1 with N-acylserines and PS 36:1, which would involve N-acylation of PS 36:1. Analysis of these potential lipid precursors found a decrease in PS 36:1 only in the PVWM (Figure 3). FA 18:0 and FA 18:1 were verified as the fatty acid substituents of both PC 36:1 (Figure 4) and PS 36:1 (Figure 4) by MS^2^. Since PS 36:1 levels were decreased in the PVWM and not in the hippocampus, our data suggest that differential regulation of PS36:1 levels in gray matter, independent of white matter, accounts for the maintenance of the steady state of this glycerophospholipid pool in the hippocampus.

We have previously reported decrements in brain ether-PS [30], which, in contrast to NAPSs, was greater in the gray matter than in the white matter in the cortex. The combined decrease in NAPS and ether-PS in LOAD has the potential to significantly negatively affect neurotransmission.

### 3.3. Diacylglycerol (DG) 36:1

**Figure 5:**
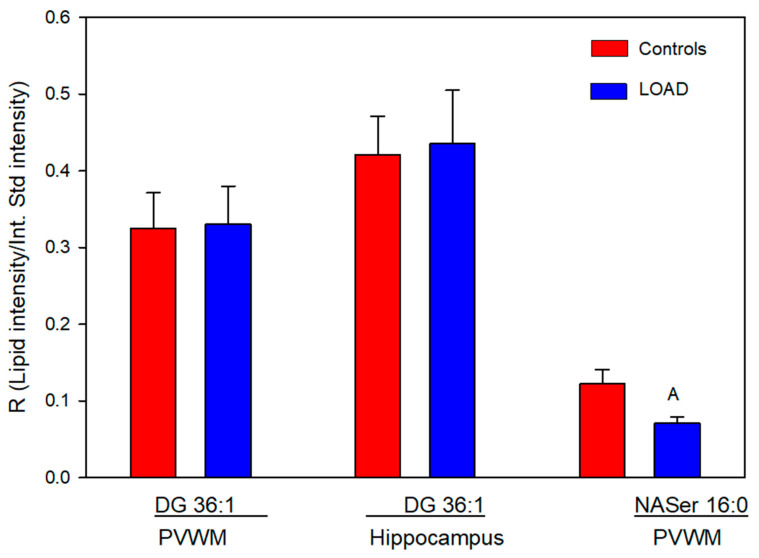
Relative levels of diacylglycerol (DG) 36:1 and N-acyl (16:0)-serine (NASer) in the PVWM and hippocampus of controls and subjects with Alzheimer’s. A (*p* = 0.043). N = 8 per group for the DGs, 7 for the Control NASer, and 6 for the LOAD NASer.

We also investigated DG 36:1, which is a potential precursor for both PC 36:1 and PS 36:1. Relative levels of this glycerolipid were unaltered in the PVWM and hippocampus (Figure 5).

### 3.4. NASer 16:0 and NASer 18:1

The N-acyl amino acids NASer 16:0 and 18:1, phospholipase D metabolic products of NAPS, were monitored at trace levels, and only NASer 16:0 was reliably measured in the PVWM. In this case relative levels were decreased by 40% in subjects with LOAD (Figure 5).

### 3.5. Lyso-NAPS 36:1

Lyso-NAPSs are isobars with the same exact masses as phosphatidylserines. Therefore, the lysoNAPSs produced from NAPS 52:1 would be lyso-NAPS 34:0 or lyso-NAPS-34:1, which are isobars of PS 34:0 and 34:1, respectively. We therefore performed MS^2^ experiments of the parent ions 762.5 and 760.5 and looked for the lysophosphatidic product ions of 423.2881 and 421.2724, respectively. We did not detect any levels of lyso-NAPS 34:0 or 34:1.

### 3.6. NASer 16:0 Phosphoric Acid

NASer16:0 phosphoric acid (NAPSer 16:0) has been reported to be a lysophosphatidic acid receptor antagonist [31]. We therefore examined our dataset to determine whether this is an endogenous phosphoaminolipid metabolic product of NAPS. NESI scans of 422.2313 did not reveal any of this lipid in the PVWM or hippocampi of controls or subjects with LOAD.

## 4. Study Limitations

The utilization of high-resolution mass spectrometry to monitor lipids, combined with structural validation, provides a high level of accuracy and reliability to the data as reflected by the limited variances.

The limitations that require consideration and hence replicative studies are the small sample sizes and the predominance of females in the LOAD group.

## 5. Discussion

**Figure 6:**
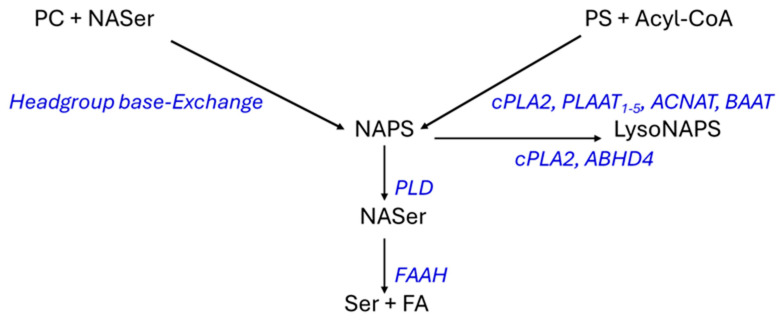
Schematic of possible synthetic pathways for NAPSs and their metabolism. cPLA2, cytosolic 238 Ca^++^-dependent phospholipase 2/N-acyltransferase (cytosol, membranes); PLAAT, phospholipase A 239 and acyltransferase (cytoplasm, nucleus); ACNAT, acyl-CoA:amino acid N-acyltransferases 240 (mitochondria); BAAT, bile acid-CoA:amino acid N-acyltransferase (cytosol, peroxisomes); PLD, 241 phospholipase D (cytosol, membranes), ABHD4, α,ꞵ-hydrolase domain-containing 4; FAAH, fatty 242 acid amide hydrolase (membrane, endoplasmic reticulum, mitochondria); PC, phosphatidylcholine; 243 PS, phosphatidylserine; NASer, N-acylserine.

NAPS 52:1 was first characterized in the bacteria *Rhodopseudomonas sphaeroides* [32] and the freshwater Bryozoan *Pectinatella magnifica* [33]. Reports of NAPS 52:1 as the dominant NAPS in the mouse brain [29] and subsequently in the human brain, with elevated levels in schizophrenia [16,25] further validated this unique lipid class. The structural roles of NAPSs require further study, but evaluations of NAPSs in liposomes suggest that NAPSs might protect membrane ultrastructure from damage in membrane fusion processes [34]. NAPSs also may function as reservoirs for bioactive N-acylserines, which can be made bioavailable by phospholipase D (Figure 6). This is a subfamily of many acyl amino acids that possess complex signal transduction functions [27,35,36,37].

The biosynthetic pathways for NAPS require more validation (Figure 6) since the conjectured pathways are based solely on the more extensive research into the biosynthetic pathways for N-acylphosphatidylethanolamines (NAPEs). Direct N-acylation of phosphatidylserines can involve cytosolic Ca^++^-dependent phospholipase 2/N-acyltransferase (cPLA2); phospholipase A and acyltransferase (PLAAT); acyl-CoA:amino acid N-acyltransferases (ACNAT); and bile acid-CoA:amino acid N-acyltransferase (BAAT) as has been demonstrated for N-acylphosphatidylethanolamines and other amino acids in peroxisomes [38] and in the cytosol [36,37,39,40]. The PLAAT family are calcium-independent N-acyltrasferases that are critical in the homeostasis of membrane function in mitochondria, lysosomes, peroxisomes, and the endoplasmic reticulum [37,41].

While cPLA2 is an N-acyltransferase [42,43], it also is hydrolytic at sn-2 of the glycerol backbone in glycerophospholipids generating lyso lipids (Figure 6). Similarly, α,ꞵ-hydrolase domain-containing 4 generates lyso NAPS [42,43,44]. In addition to N-acylation, lipid remodeling, which increases with age [45], may contribute to the synthesis of N-acylglycerophospholipids like NAPS. An alternative pathway for NAPS biosynthesis has been suggested from studies of the freshwater bryozoan *Pectinatella magnifica* [33]. This is conjectured to involve headgroup Ca^++^-dependent base-exchange [46,47,48], in this case, between a phosphatidylcholine headgroup and N-acylserine (Figure 6).

With regard to the relative roles of these two biosynthetic pathways, we noted no alterations in phosphatidylcholine levels in the PVWM or hippocampus in subjects with LOAD, but there was a decrease in phosphatidylserine in the PVWM. Similarly, N-acylserine 16:0 was decreased in the PVWM. These data may suggest that N-acylation of phosphatidylserines may be the dominant route for NAPS synthesis in the human brain, consistent with the major role of PLAAT in organelle stability [41]. While the monitored levels of N-acylserines for this route may be limiting, it is important to maintain low levels of these signaling lipids. N-Acylserines are released from NAPSs by phospholipase D and can be further regulated by metabolism to serine and a fatty acid via fatty acid amide hydrolase [36,49,50] (Figure 6).

The supply of N-acylserine also is an area that requires more definition. Currently no specific serine N-acyltransferase has been reported. In the case of glycine N-acyltransferase (EC 2.3.1.13), which is present in human mitochondria, endoplasmic reticulum, and cytosol [51], serine is a poor substrate [52].

Since there are no therapeutics available for managing oligodendrocyte/myelin function, it is important to define whether myelin dysfunction is secondary to amyloid deposition or whether myelin dysfunction contributes to LOAD pathogenesis as has recently been suggested [6]. Of significance to this discussion are the observations that exercise [53,54,55] and exercise combined with improved lifestyle [56] can prevent or delay the onset of AD. Furthermore, these positive actions of exercise on cognition may be the result of exercise promoting oligodendrocyte proliferation and myelin preservation in the elderly [55,57,58].

## 6. Conclusions

Myelin serves a key role in neuronal connectivity via support of efficient neurotransmission. Our lipid data, along with histology, proteomics, and imaging data support myelin disruption in LOAD. The decrease in white matter NAPS levels was found to be dramatic and indicates that further studies of lipid metabolism in white matter are warranted to understand the impact of these changes.

## Figures and Tables

**Table 1:** Demographics of the subjects examined in this study.

Age	Gender	Race	PMI (h)	
A. Controls			
57	M	Black or African American	31.95
64	M	White	10.4
97	F	White	3.3
85	F	White	8
58	M	Not Reported	12.3
54	M	White	8.4
95	F	White	10
67	M	White	16.3
B. LOAD			
85	F	Black or African American	48.5
89	F	White	3.3
90	F	Not Reported	15.5
83	F	White	50.1
92	M	White	4.3
83	F	White	19.9
92	F	White	2.3
89	F	White	3.6

**Table 2:** Ions extracted from the non-targeted analysis and monitored in the selected reaction monitoring (SRM) of N-acylserines. PG, phosphatidylglycerol; NAPS, N-acylphosphatidylserine; PS, phosphatidylserine; PC, phosphatidylcholine; DG, diacylglycerol; Ser-16:0, N-palmitoyl serine; Ser-18:1, N-oleoyl serine; Ser, serine; FA, fatty acid.

Lipid	Exact Mass	Ions
[^2^H_31_]PG 34:1	779.72001	[M-H]^−^: 778.7127
NAPS 52:1	1027.78165	[M-H]^−^: 1026.7743
NAPS 54:2	1053.7973	[M-H]^−^: 1052.7900
PS 36:1	789.55198	[M-H]^−^: 788.5447
PC 36:1	787.6091	[M + Cl]^−^: 822.5789
[^2^H_54_]PC 28:0	729.8257	[M + H]^+^: 730.8329
DG 36:1	622.5536	[M + NH_4_]^+^: 650.58745
Ser-16:0	343.27225	[M-H]^−^: 342.26 → Ser (104.0353/74.02475)
Ser 18:1	369.28790	[M-H]^−^: 368.28 → Ser (104.0353/74.02475)
[^2^H_31_]PG 34:1	779.72001	[M-H]^−^: 778.71 → FA 18:1 (281.2486)

## Data Availability

All data is included in the manuscript and Supplementary Excel file.

## Supplementary Materials

The following supporting information can be downloaded at https://www.mdpi.com/article/10.3390/life16010022/s1: Table S1: Excel spreadsheet of all lipid data.
