# Supplementary material for: White Matter N-Acylphosphatidylserines (NAPSs) and Myelin Dysfunction in Late-Onset Alzheimer’s Disease (LOAD): A Pilot Study
Source: Life (Basel). 2025 Dec 23;16(1):22. doi: 10.3390/life16010022 (PMC12842742; doi:10.3390/life16010022)
Supplement: Supplementary file 1 [file life-16-00022-s001.zip › life-4051136-supplementary.pdf]

Supplementary Table: All Raw Data

Supplementary Table

	NAPS 52:1		NAPS 54:2		NAPS 52:1		NAPS 54:2	
	Hippo-Con	Hippo-LOAD	Hippo-Con	Hippo-LOAD	PVWM-Con	PVWM-LOAD	PVWM-Con	PVWM-LOAD
	5.219069923	2.277761219	1.542741203	0.742045838	12.40354924	3.334069657	3.348428011	1.179392889
	4.409418316	2.764686536	1.850557475	0.960383445	9.828239163	4.861354514	3.765561939	2.28473632
	4.324425232	3.134765172	1.656847253	1.168356839	10.95999396	4.391932908	3.48798654	2.189784998
	4.485775267	3.171325432	1.822284975	1.30482661	12.26543004	5.18033021	4.411522828	2.127209696
	5.688861443	3.199542854	1.947051865	0.981802517	13.28798424	4.386415461	2.964261629	2.561910348
	4.797355785	2.366756877	1.697015323	0.874696788	19.80298875	6.479341052	5.458890035	2.217598935
	4.720584154	1.973741117	1.588112601	0.801805389	11.56622364	5.249896879	3.883248735	1.981366036
	5.199439097	2.416825858	1.904391874	0.998246344	10.40017149	3.889747934	3.711126284	1.37595837
**Mean**	**4.856**	**2.663**	**1.751**	**0.979**	**12.564**	**4.722**	**3.879**	**1.990**
**SD**	**0.476**	**0.471**	**0.151**	**0.186**	**3.135**	**0.959**	**0.765**	**0.472**
**RSD**	**9.80**	**17.68**	**8.60**	**18.96**	**24.95**	**20.31**	**19.71**	**23.72**
**N**	**8**	**8**	**8**	**8**	**8**	**8**	**8**	**8**
**SEM**	**0.168**	**0.166**	**0.053**	**0.066**	**1.108**	**0.339**	**0.270**	**0.167**
**% Control**		**54.85**		**55.91**		**37.58**		**51.30**

t-Test: Two-Sample Assuming Equal Variances

	Variable 1	ariable 2
Mean	4.855616152	2.663
Variance	0.226325296	0.222
Observations	8	8
Pooled Varianc	0.223950054	
Hypothesized M	0	
df	14	
t Stat	9.265785236	
P(T<=t) one-tail	1.19092E-07	
t Critical one-tai	1.761310136	
P(T<=t) two-tail	2.38184E-07	
t Critical two-tai	2.144786688	

t-Test: Two-Sample Assuming Equal Variances

	Variable 1	ariable 2
Mean	1.751125321	0.979
Variance	0.02266309	0.034
Observations	8	8
Pooled Varianc	0.028557845	
Hypothesized M	0	
df	14	
t Stat	9.137840153	
P(T<=t) one-tail	1.40982E-07	
t Critical one-tai	1.761310136	
P(T<=t) two-tail	2.81965E-07	
t Critical two-tai	2.144786688	

t-Test: Two-Sample Assuming Equal Variances

	Variable 1	Variable 2
Mean	12.56432257	4.721636077
Variance	9.825276168	0.919566185
Observations	8	8
Pooled Varianc	5.372421176	
Hypothesized M	0	
df	14	
t Stat	6.767212654	
P(T<=t) one-tail	4.53186E-06	
t Critical one-tai	1.761310136	
P(T<=t) two-tail	9.06372E-06	
t Critical two-tai	2.144786688	

t-Test: Two-Sample Assuming Equal Variances

	Variable 1	Variable 2
Mean	3.87887825	1.989745
Variance	0.584549321	0.222671
Observations	8	8
Pooled Varianc	0.403610218	
Hypothesized M	0	
df	14	
t Stat	5.947186814	
P(T<=t) one-tail	1.78242E-05	
t Critical one-tai	1.761310136	
P(T<=t) two-tail	3.56484E-05	
t Critical two-tai	2.144786688	

	PS 36:1		PS 36:1		PC 36:1		PC 36:1	
	WM-Con	WM-LOAD	Hippo-Con	Hippo-LOAD	Hippo-Con	Hippo-LOAD	WM-Con	WM-LOAD
	23.13513333	5.84616785	8.940408478	7.25695086	1.796508288	1.463351793	2.309915611	3.783157376
	17.82701057	11.38810258	9.586244656	8.014599723	1.922164535	2.494389479	3.54837334	1.777635206
	17.52817747	11.57586673	10.82013474	7.740146072	1.881043012	1.368089809	2.302836084	1.882757581
	20.61618579	6.493518885	14.01501592	7.110213025	1.989802448	2.036417	2.438493251	1.643227989
	12.89775149	9.923725339	6.638334572	16.92106312	1.385644365	2.789708701	3.434205705	1.42063018
	20.01969793	14.91761876	8.173749963	11.92060011	2.975015227	1.868726395	1.868726395	2.455684652
	13.83179773	22.46863382	13.79655622	11.67652983	1.472100639	1.735078498	1.735078498	3.149257364
	15.86601295	6.112431346	9.112566243	7.189984585	1.578419018	1.18252665	2.695936882	3.053762688
	**17.715**	**11.091**	**10.135**	**9.729**	1.8751	1.8673	2.5417	2.3958
**Mean**	**3.482**	**5.596**	**2.613**	**3.518**	0.4959	0.5570	0.6610	0.8529
**SD**	**19.65**	**50.46**	**25.78**	**36.16**	26.4466	29.8275	26.0074	35.5988
**RSD**	**8**	**8**	**8**	**8**	8	8	8	8
**N**	**1.231**	**1.979**	**0.924**	**1.244**	0.175326	0.196917	0.233709	0.301532
**SEM**		**62.61**		**95.99**		**99.58**		**94.26**
**% Control**								

t-Test: Two-Sample Assuming Equal Variances

	Variable 1	ariable 2
Mean	17.71522091	11.09
Variance	12.12252882	31.32
Observations	8	8
Pooled Varianc	21.71917159	
Hypothesized M	0	
df	14	
t Stat	2.84288328	
P(T<=t) one-tail	0.006515773	
t Critical one-tai	1.761310136	
P(T<=t) two-tail	0.013031546	
t Critical two-tai	2.144786688	

t-Test: Two-Sample Assuming Equal Variances

	Variable 1	
Mean	10.13537635	ariable 2
Variance	6.825378415	9.729
Observations	8	12.38
Pooled Varianc	9.600698828	8
Hypothesized M	0	
df	14	
t Stat	0.262459581	
P(T<=t) one-tail	0.398394887	
t Critical one-tai	1.761310136	
P(T<=t) two-tail	0.796789774	
t Critical two-tai	2.144786688	

	DG 36:1		DG 36:1		NASer-16:0	
	Hippo-Con	Hippo-LOAD	WM-Con	WM-LOAD	WM-Con	WM-LOAD
	0.501794831	0.270859537	0.280812334	0.517433224	0.169259333	0.081098152
	0.503702747	0.605767044	0.563088503	0.590486958	0.194789469	0.09258289
	0.373391282	0.204814932	0.675319061	0.28757301	0.086875427	0.031241713
	0.330688799	0.195079479	0.294538054	0.234769032	0.051732191	0.072928214
	0.257254588	0.437164403	0.389409859	0.605767044	0.150690695	0.067554117
	0.230983818	0.381454433	0.503702747	0.751298267	0.104177061	0.081064941
	0.131918435	0.34337725	0.263123853	0.185647749	0.09669301	
	0.270859537	0.202540856	0.403959758	0.310383087		
	0.3251	0.3301	0.4217	0.4354		
**Mean**	0.1305	0.1434	0.1481	0.2069	0.1220	0.0711
**SD**	40.1436	43.4419	35.1190	47.5218	0.0508	0.0213
**RSD**	8	8	8	8	41.6294	29.9466
**N**	0.046137	0.050705	0.052366	0.073157	7	6
**SEM**		**101.56**		**103.24**	0.019201	0.008690
**% Control**						**58.25**

t-Test: Two-Sample Assuming Equal Variances

	Variable 1	Variable 2
Mean	0.122031026	0.071078338
Variance	0.002580717	0.000453075
Observations	7	6
Pooled Varianc	0.001613607	
Hypothesized M	0	
df	11	
t Stat	2.279928384	
P(T<=t) one-tail	0.021770817	
t Critical one-tai	1.795884819	
P(T<=t) two-tail	0.043541635	
t Critical two-tai	2.20098516	
